# Enhanced Electrochemiluminescence of Luminol and-Dissolved Oxygen by Nanochannel-Confined Au Nanomaterials for Sensitive Immunoassay of Carcinoembryonic Antigen

**DOI:** 10.3390/molecules29204880

**Published:** 2024-10-15

**Authors:** Weibin Li, Ruliang Yu, Fengna Xi

**Affiliations:** 1 Shanxi Bethune Hospital, Shanxi Academy of Medical Sciences, Tongji Shanxi Hospital, Third Hospital of Shanxi Medical University, Taiyuan 030032, China; liweibin@sxbqeh.com.cn; 2 College of Chemistry and Chemical Engineering, Zhejiang Sci-Tech University, Hangzhou 310018, China; 202230107422@mails.zstu.edu.cn

**Keywords:** electrochemiluminescence immunosensor, confined Au nanomaterials, luminol, dissolved oxygen (O_2_), carcinoembryonic antigen

## Abstract

Simple development of an electrochemiluminescence (ECL) immunosensor for convenient detection of tumor biomarker is of great significance for early cancer diagnosis, treatment evaluation, and improving patient survival rates and quality of life. In this work, an immunosensor is demonstrated based on an enhanced ECL signal boosted by nanochannel-confined Au nanomaterial, which enables sensitive detection of the tumor biomarker—carcinoembryonic antigen (CEA). Vertically-ordered mesoporous silica film (VMSF) with a nanochannel array and amine groups was rapidly grown on a simple and low-cost indium tin oxide (ITO) electrode using the electrochemically assisted self-assembly (EASA) method. Au nanomaterials were confined in situ on the VMSF through electrodeposition, which catalyzed both the conversion of dissolved oxygen (O_2_) to reactive oxygen species (ROS) and the oxidation of a luminol emitter and improved the electrode active surface. The ECL signal was enhanced fivefold after Au nanomaterial deposition. The recognitive interface was fabricated by covalent immobilization of the CEA antibody on the outer surface of the VMSF, followed with the blocking of non-specific binding sites. In the presence of CEA, the formed immunocomplex reduced the diffusion of the luminol emitter, resulting in the reduction of the ECL signal. Based on this mechanism, the constructed immunosensor was able to provide sensitive detection of CEA ranging from 1 pg·mL^−1^ to 100 ng·mL^−1^ with a low limit of detection (LOD, 0.37 pg·mL^−1^, S/N = 3). The developed immunosensor exhibited high selectivity and good stability. ECL determination of CEA in fetal bovine serum was achieved.

## 1. Introduction

Cancer is one of the major global health issues posing a severe threat to human health. Cancer causes severe physical pain and functional impairment and leads to psychological stress, economic burden, and family distress [1]. Early diagnosis is crucial for improving patient survival rates and quality of life [2]. In the early stages of cancer, when the cancer cells have not widely spread, treatments are more effective and offer more options such as surgery, radiotherapy, and chemotherapy. Moreover, early diagnosis can significantly increase the cure rate, reduce treatment costs, and alleviate patients’ physical suffering. Therefore, promoting cancer screening and improving early diagnosis rates are key to cancer prevention and control. Tumor markers are substances (e.g., proteins, enzymes, hormones, or gene fragments) produced in the presence of a tumor, found in tumor cells or the host’s body, and commonly present in blood, urine, or other body fluids [3,4]. Detection of tumor markers aids in early cancer diagnosis, disease monitoring, and treatment evaluation, improving patient survival rates and quality of life [5,6,7]. For instance, carcinoembryonic antigen (CEA) is a widely used tumor marker, initially having been discovered in the blood of colon cancer patients. CEA is a protein produced during fetal development, with levels significantly decreasing after birth [8,9]. However, in certain types of cancer, CEA levels can rise abnormally. Testing CEA is significant for diagnosing and monitoring cancers such as colon cancer, pancreatic cancer, breast cancer, and lung cancer [10,11,12]. By measuring CEA levels in the blood, doctors can evaluate the presence of tumors and the effectiveness of treatments, as well as monitor disease progression and recurrence. Thus, CEA has become an important tool in cancer management, helping to improve patient survival rates and quality of life. Therefore, sensitive and convenient detection of CEA in serum is of great significance.

To date, immunoassay is commonly used for CEA detection [13,14,15]. Immunoassay utilizes the specific binding between antigens and antibodies to establish a highly selective analytical method [16,17]. By forming immunocomplexes between antibodies and their corresponding antigens (analytes), quantitative detection of the tumor marker is achieved [18]. Depending on the detection signals, the main techniques include electrochemical methods, fluorescence analysis, enzyme-linked immunosorbent assay (ELISA), and radioimmunoassay [19,20]. However, the sensitivity of ELISA is relatively low, and radioimmunoassay involves the use of radioactive substances. In recent years, electrochemiluminescence (ECL), also known as electrogenerated chemiluminescence, has gained attention as it combines the advantages of chemiluminescence and electrochemistry [21,22]. By applying a specific voltage to trigger an electrochemical reaction at the electrode surface, electrochemically generated species are produced that undergo electron transfer to form an excited state and then emit light when returning to the ground state [23,24,25]. Unlike chemiluminescence, the ECL reaction is triggered by an electrochemical process, granting it higher controllability. Compared with electrochemical analysis methods, ECL introduces a photomultiplier tube (PMT) during signal acquisition, which amplifies the light signal, thereby significantly enhancing detection sensitivity. Additionally, compared with fluorescence analysis, ECL does not require an external light source for excitation, resulting in a lower detection background and a simpler instrument structure. Thus, ECL immunoassay is a promising method for tumor marker detection, offering high sensitivity, simple equipment, convenient operation, and rapid analysis. However, existing commercial ECL immunoassays use avidin labeling and solid-phase magnetic microspheres [26,27], which both still face challenges such as expensive instruments and reagents, as well as the need for specialized skill. Developing convenient and low-cost ECL immunoassays using label-free antibody is highly desirable.

The performance of ECL immunosensors critically depends on the emitter [28]. In addition to a few emerging nanomaterials, ECL emitters are primarily categorized into inorganic and organic types. Inorganic ECL emitters, such as the metal complex tris(bipyridine)ruthenium (Ru(bpy)_3_^2+^), are widely used due to their excellent luminescence performance and stability [29,30,31]. However, Ru(bpy)_3_^2+^ is relatively expensive. Organic ECL emitters include compounds like luminol and acridinium esters [32]. Among these compounds, luminol, in particular, can generate stable luminescent signals at low voltage and is cost-effective. For luminol ECL systems, two key reactions are typically involved—the oxidation of luminol and the generation of reactive oxygen species (ROS). Under alkaline conditions, luminol can more effectively react with ROS to generate intermediates, resulting in stronger luminescence signals [33]. In neutral conditions, these reaction efficiencies are lower, thus reducing ECL efficiency and intensity. However, alkaline conditions are not suitable for the immunoassay of tumor markers. Although hydrogen peroxide (H_2_O_2_) can act as a co-reactant to generate ROS, its poor stability at room temperature limits the application of the luminol–H_2_O_2_ system. Alternatively, dissolved oxygen (O_2_) in the electrolyte is considered an ideal endogenous ROS source due to its good stability and non-toxicity. In the luminol–O_2_ system, ROS is first produced through the oxygen reduction reaction (ORR) and then reacts with electrochemically oxidized luminol to generate an excited state, resulting in the emitting of ECL. However, the efficiency of converting dissolved oxygen into ROS is low [33]. Therefore, improving the efficiency of oxygen conversion to ROS and the luminescence efficiency of luminol under neutral conditions can effectively enhance the performance of luminol–O_2_-based ECL immunoassays.

Recently, it has been proven that introducing efficient co-reactant promoters can significantly enhance ECL efficiency and improve the sensitivity and stability of biosensors. Among these promoters, noble metal nanoparticles as co-reactant promoters for the luminol–O_2_ system exhibit remarkable advantages [34,35,36,37]. Noble metals such as platinum (Pt) and gold (Au) possess high surface areas, unique electronic properties, and high catalytic activity [38,39,40], which can catalyze both the generation of more ROS or the oxidation of luminol, thereby enhancing the ECL intensity of the luminol–O_2_ system. Incorporating porous materials on electrode surfaces to confine or immobilize noble metal nanoparticles can effectively enhance the activity and stability of these nanoparticles [41,42]. Vertically-ordered mesoporous silica film (VMSF) has gained considerable attention. VMSF features a high-density, uniform nanochannel array (typically 2–3 nm) and a high specific surface area, offering potential for ECL immunosensor construction [43,44,45]. High-density nanochannels ensure the diffusion of small ECL emitters, while the nanochannels can also confine or immobilize noble metal nanoparticles as co-reactant promoters [46,47]. VMSF-modified electrodes can also eliminate or reduce the effects of complex matrices [48,49,50,51]. Due to their size-selective screening effect, ultra-small nanochannels prevent macromolecules such as proteins, DNA, and particulates from entering and contaminating the electrode surface [52,53,54,55,56]. A negatively charged surface is formed by the ionization. Additionally, the outer surface can be used for antibody immobilization, creating an immunorecognition interface [57,58,59,60]. Therefore, VMSF exhibits great potential for a high-performance ECL immunosensing system for CEA detection based on the luminol–dissolved oxygen system.

In this study, an ECL immunosensor was developed by constructing a layer of vertically-ordered mesoporous silica film with amine groups (NH_2_-VMSF) on the surface of an indium tin oxide (ITO) electrode, followed by confining Au nanomaterials within the nanochannels. An immunorecognition interface was then fabricated on the outer surface of the NH_2_-VMSF. Sensitive detection of CEA in serum samples, with a wide linear range, was achieved using luminol as the ECL emitter, dissolved oxygen as the endogenous co-reactant, and confined nanomaterials as the co-reactant promoters to significantly enhance the ECL signal of the electrode. The easy fabrication and high performance of the fabricated ECL immunosensor shows great potential in convenient and sensitive detection of the CEA tumor marker.

## 2. Results and Discussion

### 2.1. Strategy for the Construction of Immunosensor

**Figure 1:**
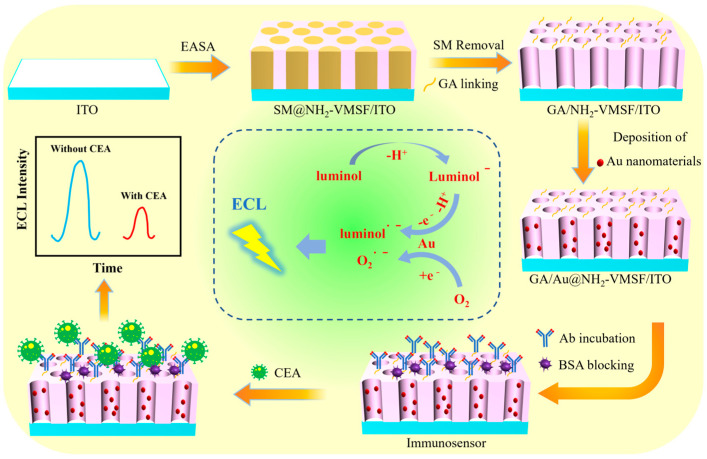
Schematic illustration for the fabrication of the ECL immunosensor for sensitive detection of CEA based on enhanced ECL by nanochannel-confined Au nanomaterials and an immunorecognition interface fabricated on the outer surface of NH_2_-VMSF.

As shown in Figure 1, an immunosensor was constructed based on amino-modified VMSF (NH_2_-VMSF) for highly sensitive ECL detection of CEA. Luminol was used as the ECL emitter; dissolved oxygen served as the endogenous co-reactant; and gold nanomaterials confined within the nanochannels acted as co-reactant accelerators to enhance the ECL signal. To reduce the cost of the immunosensor, inexpensive and readily available indium tin oxide (ITO) conductive glass was used as the supporting electrode. Using the EASA method, VMSF was quickly grown on the ITO surface within seconds to tens of seconds. Specifically, the EASA method induces a pH gradient in situ at the electrode surface by applying a negative voltage to electrolyze water and then promotes the assembly of the surfactant template and the sol–gel reaction of orgasiloxanes. By adding an amino-functionalized orgasiloxanes to the precursor solution, amino-modified VMSF could be grown in one step. The amino groups in NH_2_-VMSF serve dual functions. On the one hand, the amino groups on the outer surface can be derivatized with aldehyde groups for covalent antibody immobilization, while the amino groups within the nanochannels can act as anchoring sites for the growth of gold nanomaterials in situ.

Glutaraldehyde (GA), a bifunctional reagent, was used to derivatize the amino groups, creating an aldehyde-functionalized surface for the covalent immobilization of CEA recognition antibodies (Ab). To prevent the GA from cross-linking amino groups inside the nanochannels, which could affect mass transfer and gold nanomaterial growth, the glutaraldehyde was linked to the NH_2_-VMSF surface before removing the micelles. At this stage, the nanochannels contained a template of surfactant micelles (SMs), blocking the nanochannels, so aldehyde derivatization only occurred on the outer surface of the NH_2_-VMSF. The SMs could then be easily removed by stirring the electrode in a 0.1 M HCl–ethanol solution, resulting in a film-modified electrode with an open nanochannel array and an aldehyde-functionalized surface. After covalent immobilization of Ab and blocking non-specific sites with BSA, the immunosensor was fabricated.

Gold nanomaterials confined within the nanochannels can act as co-reactant accelerators for the endogenous co-reactant—dissolved O_2_—catalyzing both the production of reactive oxygen species (ROS) and the oxidation of luminol, as well as significantly enhancing the ECL intensity of luminol. When the immunorecognition interface specifically captures CEA, an immunocomplex is formed, reducing the diffusion of luminol and decreasing the ECL signal. Based on this mechanism, sensitive ECL detection of CEA can be achieved.

### 2.2. Characterization of Electrode Modified by NH_2_-VMSF

**Figure 2:**
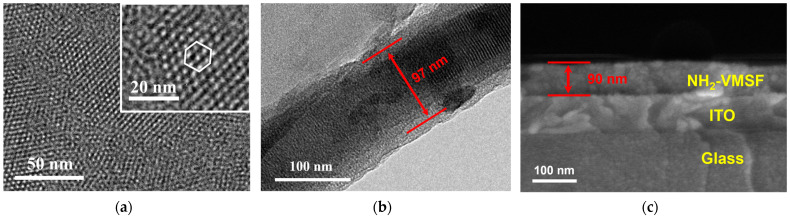
(**a**) TEM image of the top-view surface of NH_2_-VMSF. Inset is the corresponding TEM image at high magnification. The hexagon represents six adjacent nanochannels. (**b**) TEM image of the cross-section of NH_2_-VMSF. (**c**) SEM image of the cross-section of the NH_2_-VMSF/ITO electrode.

The morphology of NH_2_-VMSF grown on the ITO electrode was characterized using transmission electron microscopy (TEM) and scanning electron microscopy (SEM), as shown in Figure 2. Figure 2a presents TEM images of the NH_2_-VMSF surface at different magnifications, clearly showing that NH_2_-VMSF has no cracks and continuous nanochannels. The inset reveals that the nanochannels are arranged in an orderly hexagonal pattern, with an average pore diameter of 2.2 nm, a pore density of 8.2 × 10^12^/cm^2^, and a porosity of 31%. Figure 2b shows a cross-sectional TEM image of NH_2_-VMSF with the parallel arrangement of the channels. The film thickness is approximately 90 nm. Figure 2c displays a cross-sectional SEM image of the NH_2_-VMSF/ITO electrode, showing the glass layer and ITO layers of the ITO electrode, as well as the grown NH_2_-VMSF layer from bottom to top. The NH_2_-VMSF layer is 90 nm, consistent with the TEM characterization result.

**Figure 3:**
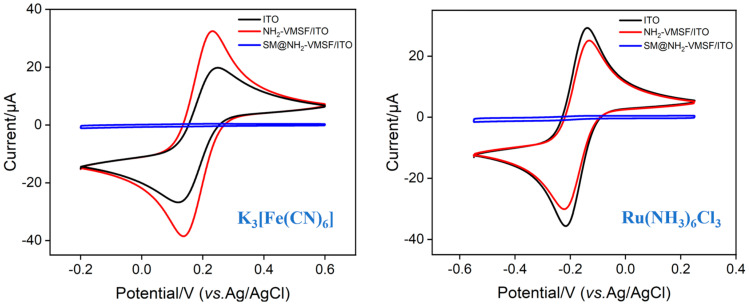
CV curves obtained from different electrodes in 0.5 mM of (**a**) K_3_[Fe(CN)_6_] and (**b**) Ru(NH_3_)_6_Cl_3_. The scanning rate is 50 mV/s.

To verify the integrity of the NH_2_-VMSF on the electrode surface and its charge properties, the electrochemical signals of different electrodes in different probe solutions were measured, as shown in Figure 3. Figure 3a displays the cyclic voltammetry (CV) curves for the negatively charged probe [Fe(CN)_6_]^3−^ on the ITO electrode, NH_2_-VMSF/ITO electrode, and SM@NH_2_-VMSF/ITO electrode with micelles in the nanochannels, respectively. Compared with the remarkable redox signal detected on the ITO electrode, almost no signal was observed on the SM@NH_2_-VMSF/ITO electrode. This is attributed to the presence of SMs that block the nanochannels, preventing the redox probe from reaching the electrode surface. This result also confirms the completeness of NH_2_-VMSF, as any crack in the film would allow the redox probe to reach the underlying electrode surface, generating a redox signal. Additionally, compared with the ITO electrode, the NH_2_-VMSF/ITO electrode, with open nanochannels after SM removal, exhibited higher electrochemical signals. This is due to the amino groups on the NH_2_-VMSF becoming protonated in the measuring medium, creating numerous positive sites that strongly attract the negatively charged [Fe(CN)_6_]^3−^ probe, enhancing its electrochemical signal. When the negatively charged probe was replaced with the positively charged Ru(NH_3_)_6_]^3+^, as shown in Figure 3b, almost no signal was observed on the SM@NH_2_-VMSF/ITO electrode, further verifying the integrity of the thin film. Notably, the peak current signal on the NH_2_-VMSF/ITO electrode decreased compared with the ITO electrode, contrary to the results with the negatively charged probe. This is because the protonated amino sites in NH_2_-VMSF electrostatically repel Ru(NH_3_)_6_]^3+^, leading to a reduction in peak current. These results confirm the integrity and charge-selective permeability of NH_2_-VMSF.

### 2.3. Characterization of Confined Au Nanomaterials

**Figure 4:**
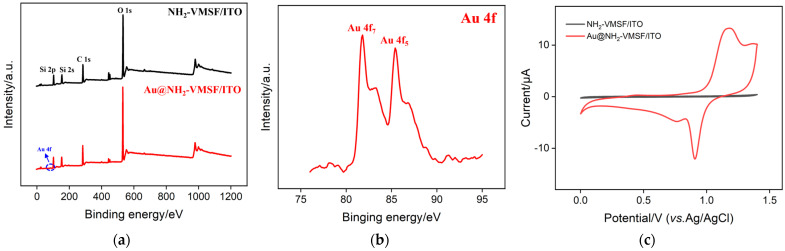
(**a**) XPS survey spectra obtained from the NH_2_-VMSF/ITO and Au@NH_2_-VMSF/ITO electrodes. (**b**) High-resolution Au 4f spectra obtained from the Au@NH_2_-VMSF/ITO electrode. (**c**) CV curves obtained from the NH_2_-VMSF/ITO or Au@NH_2_-VMSF/ITO electrode in 0.5 M of H_2_SO_4_.

To verify the successful modification of the nanochannels with Au nanomaterials, the electrode was characterized using X-ray photoelectron spectroscopy (XPS), as shown in Figure 4a. Compared with the NH_2_-VMSF/ITO electrode, the electrode modified with gold nanomaterials (Au@NH_2_-VMSF/ITO) exhibited distinct Au characteristic peaks ranging from 80–85 eV, indicating the presence of gold element on the electrode. This was further confirmed by the high-resolution Au 4f spectrum, as shown in Figure 4b, demonstrating that Au nanomaterials were successfully modified within the nanochannels. Additionally, cyclic voltammetry (CV) tests were conducted in sulfuric acid solution (H_2_SO_4_) using the electrodes before and after depositing the gold nanomaterials. As shown in Figure 4c, the deposited electrode exhibited a remarkable oxidation peak near 1.0 V and a significant reduction peak near 0.9 V, compared with that of the NH_2_-VMSF/ITO electrode. The oxidation peak at 1.1 V was attributed to the formation of Au oxide, and the reduction peak at 0.9 V was mainly attributed to the subsequent reduction of Au oxide. This is the characteristic electrochemical signal attributed to the redox reactions of the gold nanomaterials on the electrode surface. As the ITO electrode cannot undergo TEM analysis, Au@NH_2_-VMSF must be scraped from the ITO electrode using a blade to perform TEM analysis. After ultrasonic dispersion, the solution is dropped onto a copper grid for TEM observation. During this process, the gold nanomaterials are lost from the nanochannels. Thus, SEM characterization was applied to prove the deposition of gold nanomaterials within the nanochannels. As shown in the Figure S1 supporting information (SI), when the electrochemical deposition of gold nanomaterials was performed using a short time (5 s), the SEM images of the electrode before and after deposition were consistent (Figure S1a,b). However, when the deposition time of gold nanomaterials was extended to a longer time (60 s), a large number of particles appeared on the electrode surface (Figure S1c). Figure S2 presents the cross-sectional SEM image and the corresponding elemental mapping image of the Au@NH_2_-VMSF/ITO electrode, fabricated via 5 s electrodeposition of Au nanomaterials. The images reveal that the Au element is distributed within the NH_2_-VMSF layer. These results confirm that the electrodeposited Au nanomaterials are confined within the nanochannels of the NH_2_-VMSF. All above results confirm that gold nanomaterials were successfully electrodeposited within the nanochannels of NH_2_-VMSF.

### 2.4. Feasibility of Immunosensor Construction

**Figure 5:**
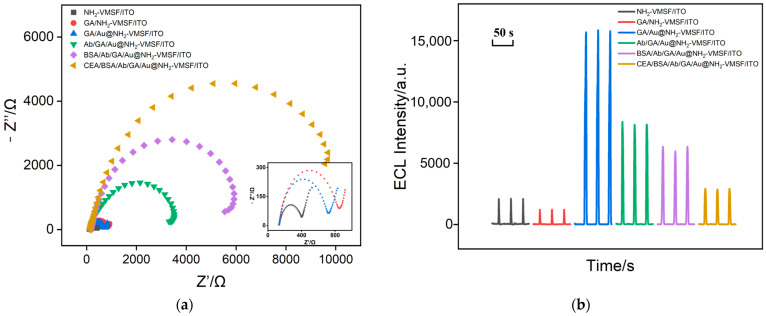
(**a**) EIS plots obtained in KCl (0.1 M) containing Fe(CN)_6_^3^/^4−^ (2.5 mM). The frequency range for EIS measurements was from 0.1 Hz to 100 kHz, with a perturbation amplitude of 5 mV. The used CEA solution was 1 ng mL^−1^ in PBS. (**b**) The ECL signal obtained from different electrodes in 100 μM of luminol. The bias of PMT was 700 V. The CV scan range was from −1.0 V to 0.8 V, with a scan rate of 100 mV/s.

Electrochemical impedance spectroscopy (EIS) was used to verify the feasibility of the immunosensor fabrication through measuring the interfacial changes of the electrodes obtained during the construction of the immunosensor. EIS plots were obtained to investigate the charge transfer resistance (Ret) of the electrodes, as shown in Figure 5a. Compared with the NH_2_-VMSF/ITO electrode (291 ± 15 Ω), the Ret increased after modification with GA (710 ± 32 Ω), possibly due to the interaction between GA and the amino groups on the electrode surface, which may slightly reduce the diffusion of [Fe(CN)_6_]^3−^. After modifying the nanochannels with Au nanomaterials, the Ret of the Au@NH_2_-VMSF/ITO electrode decreased to 594 ± 27 Ω, attributed to the high conductivity and the large specific surface area of the Au nanomaterials. When Ab was covalently immobilized on the surface of NH_2_-VMSF and blocked with BSA, the Ret of the electrode significantly increased (3554 ± 137 Ω and 6278 ± 409 Ω, respectively) due to the insulating properties and large size of the proteins reducing the diffusion of [Fe(CN)_6_]^3−^. When the target CEA was present in the solution, the Ret of the electrode further increased to 10741 ± 451 Ω, demonstrating the specific binding between the immunorecognition interface and CEA. These results confirm the effective construction of the immunosensor. Figure 5b shows the ECL signals measured from various electrodes during the construction of the immunosensor. It can be seen that the ECL signal of the electrode significantly increases when gold nanomaterials are confined within the nanochannels. The following immobilization of Ab and BSA blocking, as well as CEA binding, reduced the ECL of the electrode, owing to the decreased diffusion of luminol to the supporting electrode.

### 2.5. Mechanism of the Enhanced ECL by Confined Au Nanomaterials

**Figure 6:**
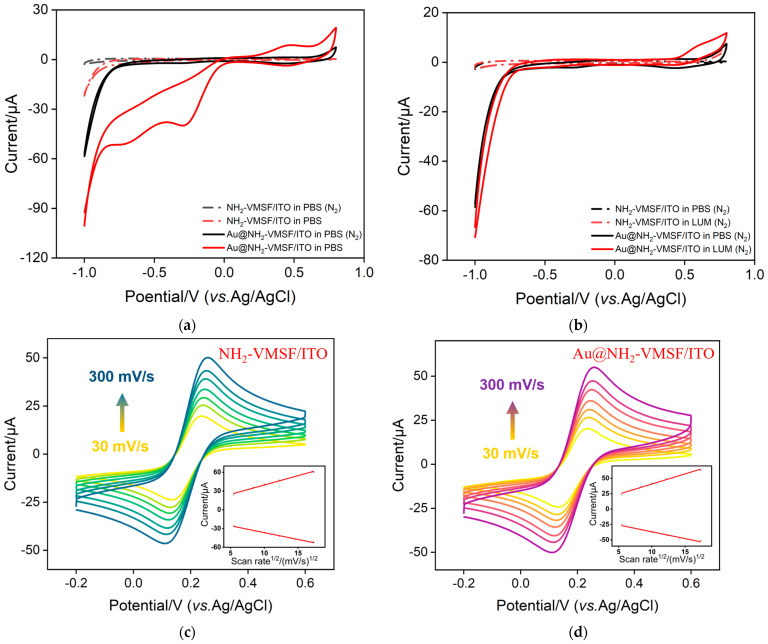
(**a**) CV curves obtained from different electrodes in the absence or presence of dissolved oxygen. (**b**) CV curves obtained from different electrodes in the absence or presence of luminol. CV curves obtained from the NH_2_-VMSF/ITO (**c**) and the Au@NH_2_-VMSF/ITO electrodes (**d**) in 0.1 M KHP (pH 4) containing 0.5 mM K_3_[Fe(CN)_6_]. The scan rate was 30, 50, 70, 100, 150, 200, and 300 mV/s, respectively. Insets are the linear relationships between the peak current and the square root of the scan rate obtained from (**c**) the NH_2_-VMSF/ITO or (**d**) the Au@NH_2_-VMSF/ITO electrode.

To investigate the ECL mechanism, CV measurements were conducted on different electrodes under various conditions, as shown in Figure 6a. When the NH_2_-VMSF/ITO electrode was immersed in a PBS solution without O_2_, no noticeable redox peaks were observed in the CV curve. However, when the electrode was immersed in an O_2_-containing PBS solution, a reduction peak appeared at −0.8 V, attributed to the reduction of water. When the Au@NH_2_-VMSF/ITO electrode was immersed in a PBS solution without O_2_, a slight oxidation peak was observed at positive potentials due to the oxidation of water on the surface of the Au nanomaterials, thus generating O_2_. When the Au@NH_2_-VMSF/ITO electrode was immersed in the O_2_-containing PBS solution, the oxidative peak of O_2_ appeared at +0.5 V, indicating the electrochemical oxidation of O_2_ catalyzed by Au nanomaterials. Additionally, a reduction peak at −0.3 V was observed, attributed to the high catalytic activity of Au nanomaterials in reducing dissolved oxygen.

To verify the catalytic effect of Au nanomaterials on luminol, CV tests were conducted on electrodes in solutions with and without luminol, as shown in Figure 6b. First, the NH_2_-VMSF/ITO electrode was immersed in PBS and in 100 μM luminol solution, revealing an oxidation peak at +0.8 V in the latter, corresponding to the luminol anion (LH^−^) losing an electron and a proton to form luminol anion radicals (L^·−^). When the electrode was replaced with the Au@NH_2_-VMSF/ITO electrode, the oxidation peak further increased, and the peak potential shifted negatively, indicating that LH^−^ lost more electrons and protons to form more L^·−^ under the catalytic effect of Au. Therefore, the modification with Au nanomaterials not only catalyzes the production of more ROS from O_2_ but also generates more luminol anion radicals, significantly enhancing the ECL signal of the luminol–dissolved oxygen system.

Given the high conductivity of Au nanomaterials, the changes in the electrochemical active area of the electrodes before and after Au nanomaterial deposition were measured, as shown in Figure 6c,d. Figure 6c present CV curves at different scan rates and corresponding linear equations of peak currents versus the square root of the scan rate for the NH_2_-VMSF/ITO electrode. The linear equation of the oxidation peak current (I_pa_) with the scan rate (v) is I_pa_ = 2.99 v^1/2^ + 9.76. According to the Randles–Sevcik equation (Ip = 2.69 × 105 AD^1/2^ n^3/2^ v^1/2^ C), the electrochemical active area of the electrode is calculated to be 0.272 cm^2^. Here, A is the electrochemical active area; D is the diffusion coefficient of the probe solution K_3_[Fe(CN)_6_], which is 6.67 × 10^−6^ cm^2^ s^−1^; n is the number of electrons transferred (n = 1 here); v is the scan rate; and C is the bulk concentration of K_3_[Fe(CN)_6_]. Figure 6d shows curves at different scan rates and corresponding linear equations for the Au@NH_2_-VMSF/ITO electrode. According to the oxidation peak current linear equation, I_pa_ = 3.29 v1/2 + 7.80, the electrochemical active area after Au nanomaterial modification is calculated to be 0.299 cm^2^. This indicates that the electrochemical active area increased by approximately 10% after Au nanomaterial modification, while the ECL signal increased by about five times, demonstrating that the main contribution of Au nanomaterial modification is catalytic effect. The good linear relationship between the peak current and the square root of the scan rate also indicates that the electrochemical process on the electrode surface is diffusion controlled.

The stability of the Au@NH_2_-VMSF/ITO electrode was also investigated. An electrode prepared by depositing gold nanomaterials on an unmodified ITO electrode (Au@ITO) using the same method was used as a control. The time-dependent ECL signal curves from continuous measurements on both electrodes are shown in Figure S3 (SI). It can be observed that the signal on the Au@ITO electrode decreases significantly with increasing measurement time, with the ECL intensity after 10 measurements being only 60% of the initial value. This is attributed to the instability of the gold nanomaterials on the surface of the ITO electrode during usage. In contrast, the ECL signal on the Au@NH2-VMSF/ITO electrode remains highly stable, which can be attributed to the confinement effect of the nanochannels, significantly enhancing the stability of the gold nanomaterials. Thus, the use of electrodeposition to directly grow gold nanomaterials within nanochannels enhances the stability of the nanomaterials.

**Figure 7:**
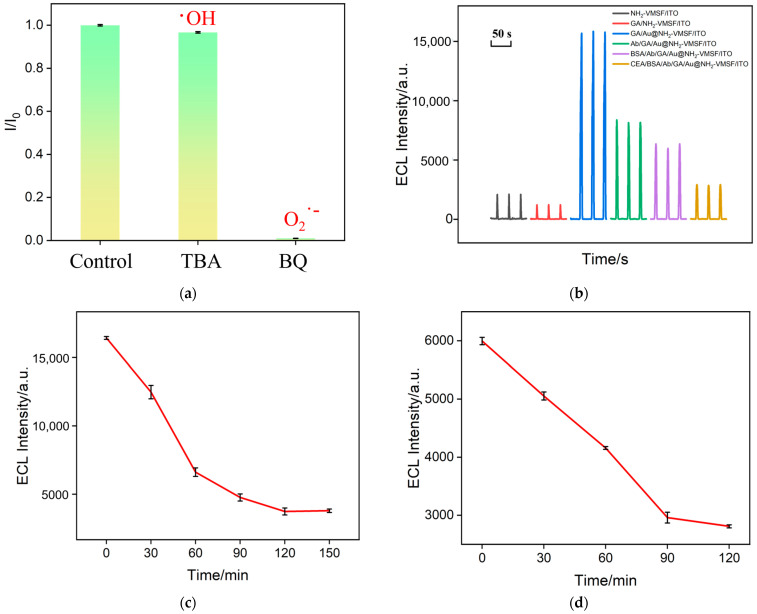
(**a**) The ratio of the ECL signal obtained from the Au@NH_2_-VMSF/ITO electrode in the presence of different radical trapping agents. The concentration of 1,4-benzoquinone (BQ) and tert-butanol (TBA) was 100 μM and 100 μg/mL, respectively. (**b**) The ECL signal obtained from the Au@NH_2_-VMSF/ITO electrode in 0.01 M PBS (pH = 7.4) solution containing 100 μM of luminol, in which Au nanomaterials were deposited for 0 s, 1 s, 2 s, 5 s, or 10 s, respectively. The concentration of the HAuCl_4_ solution was 1 mM. (**c**) The ECL signal obtained from an immunosensor fabricated using different times for Ab immobilization. (**d**) The ECL signal obtained using different times for CEA binding.

To further verify the catalytic mechanism between Au nanomaterials and luminol, and to investigate the active oxygen species generated under Au catalysis, Au@NH_2_-VMSF/ITO was investigated to the addition of tert-butanol and p-benzoquinone to quench hydroxyl radicals (OH^·^) and superoxide anion radicals (O_2_^·−^), respectively, as shown in Figure 7a. It was observed that the addition of hydroxyl radical quenchers did not significantly reduce the ECL signal of the system, indicating that superoxide anion radicals are the primary reactive species involved.

Based on the above experimental results, the possible ECL mechanism of the luminol–O_2_ system constructed in this study is demonstrated in Figure S4 (SI). First, luminol ionizes in solution, losing a proton to form singly deprotonated luminol (LH^−^); subsequently, under the catalytic effect of Au nanomaterials, LH^−^ loses an electron and a proton to form a luminol anion radical (L^·−^); simultaneously, O_2_ is reduced to a superoxide anion radical (O_2_^·−^) by gaining an electron under the catalytic effect of Au nanomaterials. The generated O_2_^·−^ reacts with L^·−^ to form 3-aminophthalate dianion excited states (AP^2−^*) and O_2_; finally, AP^2−^* returns to the ground state to become 3-aminophthalate dianion (AP^2−^), releasing energy, as well as emitting in order to produce the ECL signal.

### 2.6. Optimized Condition for the Fabrication of the Immunosensor

To achieve maximum detection sensitivity of the electrode, the deposition time of Au nanomaterials and the incubation time for antibody immobilization and CEA at the immunorecognition interface were both optimized. Figure 7b shows the optimization of the Au nanomaterial deposition time. Within the deposition time range of 1 to 5 s, the ECL signal gradually increased as the deposition time increased due to the increased deposition of Au nanomaterials. However, when the deposition time reached 10 s, the ECL signal sharply decreased, likely due to excessive deposition of Au nanomaterials leading to pore blockage, which inhibited the mass transfer of the electrochemical probe, thus reducing the ECL signal. Therefore, 5 s was selected as the optimal deposition time. Additionally, the incubation time for Ab with the aldehyde-modified outer surface during antibody immobilization was also optimized, as shown in Figure 7c. With an increasing incubation time for antibody immobilization, the number of antibodies fixed on the electrode surface increased, significantly hindering the diffusion of the luminol emitter, resulting in a gradual decrease in the ECL signal. When the incubation time reached 120 min, any further increase in the incubation time did not result in significant signal changes, indicating that the number of antibodies immobilized on the electrode surface had nearly reached saturation. Therefore, 120 min was chosen as the optimal incubation time for antibody immobilization. Similarly, the antigen incubation time was optimized, as shown in Figure 7d. When the incubation time reached 90 min, the ECL signal of the electrode stabilized, indicating that the antigen binding sites on the electrode surface were nearly saturated. Therefore, 90 min was selected as the optimal incubation time for the antigen.

### 2.7. ECL Detection of CEA

**Figure 8:**
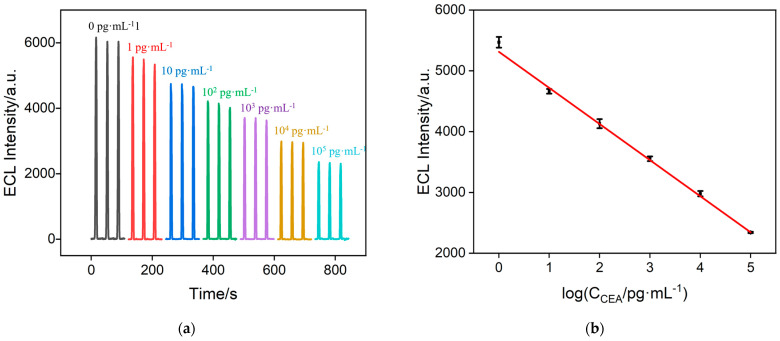

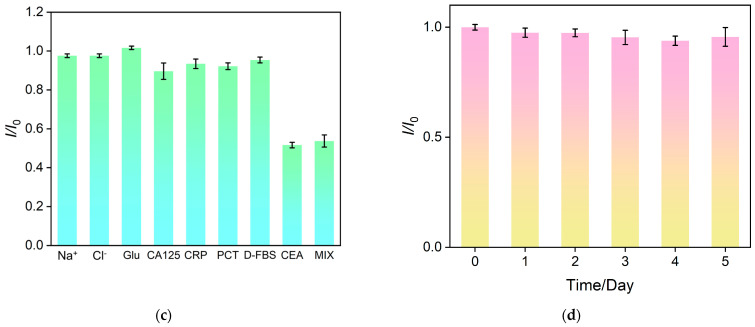
(**a**) ECL signals from the fabricated immunosensor incubated with different concentrations of CEA. (**b**) Linear calibration curve of CEA detection. (**c**) The ratio of the ECL signal of the immunosensor before (I_0_) and after (I) incubation with the different substances. The concentration of Na^+^ and Cl^−^ is 10 μM; the concentration of Glu is 20 μM; the concentration of CA125 is 200 mU mL^−1^; the concentration of CRP and PCT is 200 ng mL^–1^; the fetal bovine serum was diluted 50 times using PBS (D-FBS); and the concentration of CEA is 10 ng mL^–1^. (**d**) Stability of the immunosensor within five days.

Under the optimized detection conditions, the constructed immunosensor was incubated with different concentrations of CEA antigen, and the ECL signals of the electrode before and after CEA binding were measured, as shown in Figure 8a. It can be observed that as the concentration of CEA increases, the ECL signal of the electrode gradually decreases. This is attributed to the formation of immunocomplexes on the immunorecognition interface, which hinders the diffusion of luminol to the underlying electrode. As shown in Figure 8b, when the CEA concentration ranges from 1 pg·mL^−1^ to 100 ng·mL^−1^, the ECL signal of the electrode (I) exhibits a good linear relationship with the logarithm of the CEA concentration (logC_CEA_) (I = −592 logC + 5313, R^2^ = 0.998). The limit of detection (LOD) was calculated to be 0.37 pg·mL^−1^ based on a signal-to-noise ratio of three (S/N = 3).

### 2.8. Selectivity, Anti-Interference, and Stability of the Electrode

The selectivity and anti-interference of the constructed immunosensor were evaluated by selecting common substances that may coexist in real samples, including the most common inorganic ions in blood, such as sodium ions (Na^+^) and chloride ions (Cl^−^); small molecular metabolites commonly found in biological samples, such as glucose (Glu); and tumor markers, such as carbohydrate antigen 125 (CA125), C-reactive protein (CRP), and procalcitonin (PCT). The ratio of the ECL signal of the immunosensor before (I_0_) and after (I) incubation with these above substances (I_0_/I) is shown in Figure 8c. It can be seen that even with the concentration of other proteins being 20 times that of CEA, the ECL signals did not significantly change. Only when CEA or CEA-containing mixtures were incubated with the immunosensor did the ECL signal of the electrode change significantly, indicating that the constructed immunosensor electrode has good selectivity and anti-interference ability. Additionally, the stability of the electrode was measured. As shown in Figure 8d, the signal of the electrode remained above 94% of the initial signal after five days of storage, indicating that the electrode has good stability.

### 2.9. Real Sample Analysis

To evaluate the capability of the constructed immunosensor in detecting real samples, the content of CEA in fetal bovine serum (FBS) samples was measured using the standard addition method. Due to the high viscosity of FBS, direct measurement was not feasible. At the same time, the constructed sensor exhibited high sensitivity. Thus, FBS was diluted by a factor of 50. The results are shown in Table 1. It can be seen that the recovery for CEA detection ranged from 95.8% to 112% using the linear regression curve in PBS, with a relative standard deviation (RSD) of less than 3%. The results demonstrate that the matrix effect of the diluted FBS is very low. This could be attributed to the size-exclusion effect and the insulating properties of VMSF, which both significantly reduce matrix fouling on the underlying electrode. Thus, the constructed immunoelectrode has potential for detecting CEA in real samples.

## 3. Materials and Methods

### 3.1. Chemicals and Materials

Carcinoembryonic antigen (CEA) and its corresponding antibody (Ab) were purchased from Okay Biotechnology Co., Ltd. (Nanjing, China). Sodium dihydrogen phosphate dihydrate (NaH_2_PO_4_·2H_2_O), disodium hydrogen phosphate dodecahydrate (Na_2_HPO_4_·12H_2_O), cetyltrimethylammonium bromide (CTAB), tetraethyl orthosilicate (TEOS), potassium ferricyanide (K_3_[Fe(CN)_6_]), potassium hydrogen phthalate (KHP), 3-aminopropyltriethoxysilane (APTES), hexaammineruthenium(III) chloride (Ru(NH_3_)_6_Cl_3_), chloroauric acid trihydrate (HAuCl_4_·3H_2_O), glutaraldehyde (GA), and bovine serum albumin (BSA) were purchased from Aladdin Biochemical Technology Co., Ltd. (Shanghai, China). Fetal bovine serum was obtained from Sigma-Aldrich (Shanghai, China). Sodium nitrate (NaNO_3_), anhydrous ethanol (99.8%), and acetone were purchased from Gaoxin Fine Chemical Co., Ltd. (Hangzhou, China). All reagents were used without further purification, and all aqueous solutions were prepared with ultrapure water (18.2 MΩ·cm). ITO conductive glass (sheet resistance < 17 Ω/sq, ITO thickness: 100 ± 20 nm) was purchased from Kaivo Optoelectronic Technology Co., Ltd. (Zhuhai, China). The ITO glass was cleaned and used as the supporting electrode. Specifically, the electrode was soaked in a solution of NaOH (1 M) overnight and then ultrasonically cleaned sequentially in acetone, ethanol, and water for 10 min each.

### 3.2. Measurements and Instrumentations

All electrochemiluminescence (ECL) tests and cyclic voltammetry (CV) measurements were conducted using a three-electrode system. Briefly, a silver/silver chloride electrode (Ag/AgCl) served as the reference electrode with a saturated potassium chloride (KCl) solution as the internal reference solution. A platinum wire or platinum sheet electrode was employed as the counter electrode, and the ITO electrode—both before and after modification—was the working electrode. The ECL tests were carried out on an MPI-E II (Remex Analytical Instrument Co., Ltd., Xi’an, China) instrument, with a scan rate of 100 mV/s for CV and a photomultiplier tube (PMT) bias of 700 V. The CV tests were performed using an Autolab electrochemical workstation (PGSTAT302N, Altkirch, Freiburg, Switzerland) with a scan rate of 50 mV/s. The electrode morphology and element mapping of energy-dispersive spectroscopy were characteri, zed using a scanning electron microscope (SEM, Zeiss Gemini 300, Oberkochen, Baden-Württemberg, Germany) or a transmission electron microscope (TEM, JEM-2100, Hitachi, Tokyo, Japan). To obtain SEM samples of the electrode cross-section, a knife was used to make a scratch on the back of the electrode, and it was gently broken to expose the cross-section. The electrode surface and cross-section were coated with platinum for 60 s before SEM testing or element mapping. The accelerating voltage during the measurements was 5 kV. For the TEM samples, the NH_2_-VMSF layer was scraped from the electrode using a knife, placed in anhydrous ethanol, and ultrasonicated for uniform dispersion. Then, the solution was dropped onto a copper grid and allowed to air dry before TEM observation. The accelerating voltage for TEM observation was 200 kV.

### 3.3. Fabrication of the Immunosensor

Amino-modified VMSF—NH_2_-VMSF—was grown on the surface of the ITO electrode using the electrochemically assisted self-assembly (EASA) method [61,62]. Specifically, CTAB (1.585 g) was first added to a mixed solution of NaNO_3_ (0.1 M, 20 mL) and ethanol (20 mL) and stirred until completely dissolved. Then, APTES (318 μL) was added, and the pH was adjusted to 3.0 using concentrated hydrochloric acid. After adding TEOS (2732 μL), the mixture was stirred at room temperature for 2.5 h to prepare the precursor solution for growing NH_2_-VMSF. Subsequently, a cleaned ITO electrode was used as the working electrode, and a constant current (*j* = −0.7 mA/cm^2^) was applied for 10 s to grow NH_2_-VMSF. The resulting electrode was rinsed with ultrapure water and then aged at 120 °C for 12 h to obtain an electrode with a template of surfactant micelles (SMs) in the nanochannels, designated as SM@NH_2_-VMSF/ITO.

The amino groups on the surface of the SM@NH_2_-VMSF/ITO electrode were derivatized to aldehyde groups using glutaraldehyde (GA). Specifically, the SM@NH_2_-VMSF/ITO electrode was immersed in a 5% GA solution and incubated in the dark at 37 °C for 30 min, resulting in a GA/SM@NH_2_-VMSF/ITO electrode. Subsequently, the electrode was immersed in a 0.1 M hydrochloric acid–ethanol solution and stirred for 5 min to remove the surfactant micelles from the NH_2_-VMSF nanochannels, obtaining a modified electrode with open nanochannels (NH_2_-VMSF/ITO). Gold nanomaterials were then grown in situ in the nanochannels by electrodeposition (Au@NH_2_-VMSF/ITO). Specifically, the electrode was immersed in a 1 mM chloroauric acid solution, and a constant voltage of −0.5 V was applied for 5 s, resulting in an electrode with confined gold nanomaterials in the nanochannels, designated as the GA/Au@NH_2_-VMSF/ITO electrode.

The GA/Au@NH_2_-VMSF/ITO electrode was then immersed in a carcinoembryonic antigen (CEA) antibody solution (Ab, 10 μg/mL) and incubated at 4 °C for 120 min to obtain an immunorecognition interface, designated as the Ab/GA/Au@NH_2_-VMSF/ITO electrode. Finally, the electrode was incubated with 1% bovine serum albumin (BSA) at 4 °C for 30 min to block non-specific sites, resulting in the final immunosensor, designated as the BSA/Ab/GA/Au@NH_2_-VMSF/ITO electrode.

### 3.4. Detection of CEA

The fabricated immunosensor was incubated with a series of CEA solutions of different concentrations at 4 °C for 90 min. Then, the unbound antigens were rinsed off with ultrapure water. The ECL signals of the electrode before and after CEA binding were measured using continuous CV scanning to trigger the ECL process. The CV scan range was from −1.0 V to 0.8 V, with a scan rate of 100 mV/s. The electrolyte solution for detection was PBS (0.1 M, pH 7.4), containing (100 μM) luminol. For real sample analysis, the standard addition method was used to determine CEA in fetal bovine serum. Before measurement, the fetal bovine serum was diluted fifty times using PBS (0.1 M, pH 7.4).

## 4. Conclusions

In summary, an immunosensor was developed based on the nanochannel-confined Au nanomaterials, which amplified the ECL signal of the luminol–dissolved oxygen system in a neutral medium, achieving highly sensitive detection of CEA. The Au nanomaterials confined within the nanochannels significantly enhanced the ECL response of the luminol–O_2_ system by increasing the active surface area of the electrode, efficiently catalyzing the generation of ROS from dissolved oxygen, and catalyzing the oxidation of luminol. Additionally, the amino groups on the outer surfaces of the nanochannels were derivatized with aldehyde groups, enabling the covalent immobilization of specific recognition antibodies. When CEA was captured by the immunorecognition interface, the resulting immunocomplex hindered the diffusion of luminol to the supporting electrode, thereby reducing the ECL signal. Based on this mechanism, sensitive ECL detection of CEA was achieved. The constructed immunosensor exhibited high selectivity and stability, demonstrating potential for tumor marker detection.

## Figures and Tables

**Table 1:** Detection of CEA in fetal bovine serum by the standard addition method using the fabricated immunosensor.

Sample	Added (ng mL^−1^)	Found (ng mL^−1^)	Recovery (%)	RSD (%, n = 3)
fetal bovine serum ^a^	0.100	0.0958 ± 0.001	95.8	1.0
	1.00	1.12 ± 0.03	112	2.7
	10.0	9.77 ± 0.12	97.7	1.2

^a^ The fetal bovine serum was diluted by a factor of 50 using PBS (0.01 M, pH 7.4).

## Data Availability

The data presented in this study are available on request from the corresponding author.

## Supplementary Materials

The following supporting information can be downloaded at: https://www.mdpi.com/article/10.3390/molecules29204880/s1, Figure S1: SEM image of NH_2_-VMSF/ITO before (a) and after electrochemical deposition Au nanomaterial for 5s (b) (B) SEM image of Au@NH_2_-VMSF/ITO prepared using electrochemical deposition of Au nanomaterial for 60s (c); Figure S2: The cross-sectional SEM (left) and corresponding elemental mapping (right) images of the Au@NH_2_-VMSF/ITO electrode, fabricated *via* 5-second electrodeposition of Au nanomaterials; Figure S3: The time-dependent ECL signal curves obtained from continuous measurements on different electrodes; Figure S4: The possible ECL mechanism of the luminol-O_2_ system constructed in this study.
