# Supplementary material for: Enhanced Electrochemiluminescence of Luminol and-Dissolved Oxygen by Nanochannel-Confined Au Nanomaterials for Sensitive Immunoassay of Carcinoembryonic Antigen
Source: Molecules. 2024 Oct 15;29(20):4880. doi: 10.3390/molecules29204880 (PMC11510663; doi:10.3390/molecules29204880)
Supplement: Supplementary file 1 [file molecules-29-04880-s001.zip › molecules-3175682-supplementary.pdf]

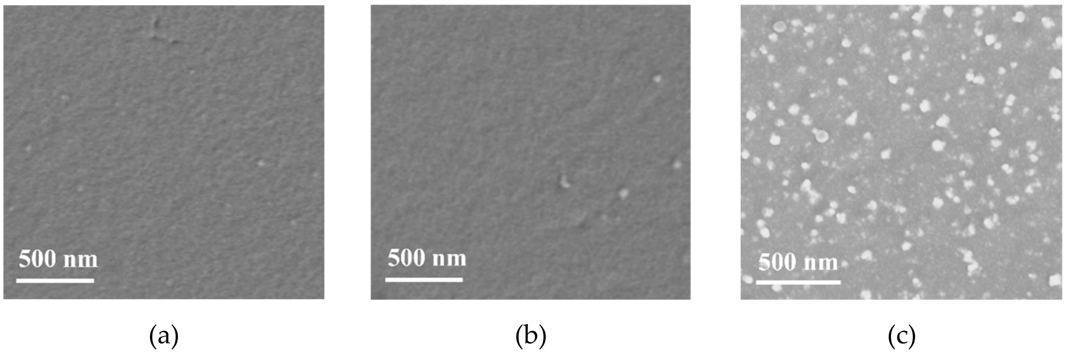

Figure S1. SEM image of NH_2-VMSF/ITO before (a) and after electrochemical deposition Au nanomaterial for 5s (b) (B) SEM image of Au@NH_2-VMSF/ITO prepared using electrochemical deposition of Au nanomaterial for 60s (c).

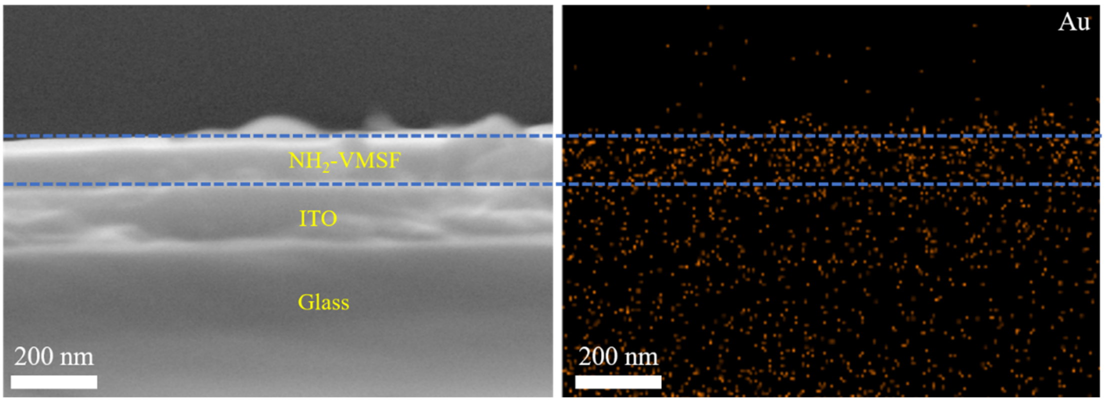

Figure S2. The cross-sectional SEM (left) and corresponding elemental mapping (right) images of the Au@NH_2-VMSF/ITO electrode, fabricated *via* 5-second electrodeposition of Au nanomaterials.

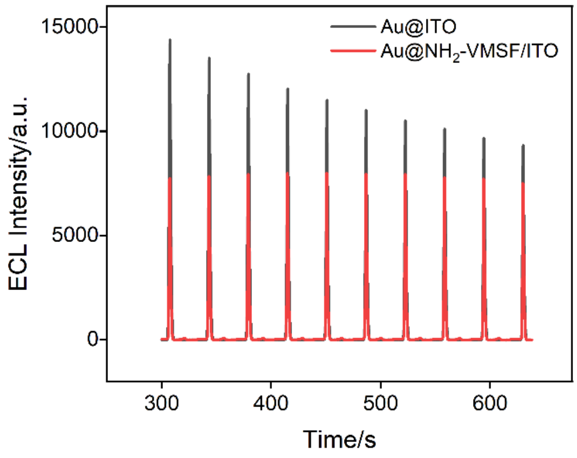

Figure S3. The time-dependent ECL signal curves obtained from continuous measurements on different electrodes.

$$LH_2 - H^+ \rightarrow LH^- \qquad (1)$$

$$LH^- - e^- - H^+ \xrightarrow{\text{Au}} L^{\bullet -} \qquad (2)$$

$$O_2 + e^- \xrightarrow{\text{Au}} O_2^{\bullet -} \qquad (3)$$

$$L^{\bullet -} + O_2^{\bullet -} \rightarrow AP^{2-*} + O_2 \qquad (4)$$

$$AP^{2-*} \rightarrow AP^{2-} + hv \qquad (5)$$

Figure S4. The possible ECL mechanism of the luminol-O_2 system constructed in this study.
